# Genetics of NLRP3 suggests lack of involvement and inefficient druggability in Parkinson’s disease

**DOI:** 10.1101/2023.09.20.23295790

**Published:** 2023-09-23

**Authors:** Konstantin Senkevich, Lang Liu, Chelsea X. Alvarado, Hampton L. Leonard, Mike A. Nalls, Ziv Gan-Or

**Affiliations:** 1. The Neuro (Montreal Neurological Institute-Hospital), McGill University, Montreal, Quebec, Canada; 2. Department of Neurology and neurosurgery, McGill University, Montréal, QC, Canada, Canada; 3. Department of Human Genetics, McGill University, Montréal, QC, Canada; 4. Center for Alzheimer’s and Related Dementias (CARD), National Institute on Aging and National Institute of Neurological Disorders and Stroke, National Institutes of Health, Bethesda, MD, USA, 20814; 5. Data Tecnica, Washington, DC, USA, 200373.; 6. DZNE Tübingen, Tübingen, Germany

**Keywords:** Parkinson’s disease, NLRP3, CASP1, genetics, inflammation

## Abstract

Activation of the NLRP3-inflammasome has been proposed to play a role in Parkinson’s disease pathogenesis based on *in vitro* and *in vivo* studies. Currently, clinical trials targeting the NLRP3 pathway in Parkinson’s disease are at early stages. However, the evidence supporting NLRP3’s involvement in Parkinson’s disease from human genetics data remains limited. In this study, we conducted comprehensive analyses of common and rare variants in genes related to the NLRP3-inflammasome in large Parkinson’s disease cohorts. Furthermore, we performed pathway-specific analyses using polygenic risk scores and studied potential causal associations using Mendelian randomization with the NLRP3 components and the cytokines released by its activation, IL-1β and IL-18. Our findings showed no associations of common or rare variants, nor of the pathway polygenic risk score for the NLRP3 inflammasome, with risk of Parkinson’s disease. Mendelian randomization analyses suggest that altering the expression of the NLRP3 inflammasome, IL-1β or IL-18, is not likely to affect Parkinson’s disease risk, age-at-onset, or progression. Therefore, our results do not support an important role for the NLRP3 inflammasome in Parkinson’s disease pathogenesis or as a strong target for drug development.

## Introduction

In recent years, activation of the nucleotide-binding oligomerization domain-, leucine-rich repeat and pyrin domain-containing 3 (NLRP3) inflammasome has been implicated in Parkinson’s disease by numerous functional studies using different models^1^. Inflammasomes are protein complexes which serve as signaling platforms for activation of immune response. The NLRP3 inflammasome comprises three main components: NLRP3 (encoded by the *NLRP3* gene), apoptosis-associated speck-like protein containing a caspase activating recruitment domain (encoded by *PYCARD*) and caspase-1 (*CASP1*). NLRP3 is expressed in microglia and when activated, it leads to secretion of the cytokines IL-1β and IL-18, which leads to neuroinflammatory response and pyroptosis^2^.

The evidence for the involvement of the NLRP3 inflammasome in Parkinson’s disease is mainly derived from *in vitro* and *in vivo* cell and animal models, by interacting with α-synuclein, mitochondria and other mechanisms. For example, early research suggested that in human monocytes, α-synuclein may directly trigger the NLRP3 inflammasome^3^. Similar results have been reported in other cell and animal models^4,5^. Other studies in cell and animal models have suggested that the NLRP3 inflammasome may be involved in toxinmediated Parkinson’s disease and that there could be an interplay between mitochondria and the NLRP3 inflammasome in Parkinson’s disease pathogenesis^6,7^. In humans, one study reported that a genetic variant in *NLRP3* may affect its expression and the risk of Parkinson’s disease^8^. Several studies in cells and postmortem brain tissues from Parkinson’s disease patients and controls reported alterations in the NLRP3 inflammasome in Parkinson’s disease^8–10^. However, there are no thorough human genetic studies of the NLRP3 inflammasome in Parkinson’s disease, although such studies can help with inferring causality. Nevertheless, there is a suggestion that the NLRP3 inflammasome may be a good target for therapeutic development in Parkinson’s disease, and several compounds targeting the NLRP3 inflammasome are in different stages of development^11^. Considering that clinical trial success rates increase significantly when supported by genetic evidence^12^, it becomes crucial to conduct thorough genetic analysis of the proposed target.

In this study, we aimed to examine whether human genetics data supports NLRP3 involvement in Parkinson’s disease and development of therapeutics targeting NLRP3 for Parkinson’s disease. We analyzed common and rare variants in the NLRP3 inflammasome components in large Parkinson’s disease cohorts, and further performed pathway specific analyses of polygenic risk scores (PRS) and Mendelian randomization (MR) analyses. Our results do not support an important role for the NLRP3 inflammasome in Parkinson’s disease nor its being a good target for therapeutic development in sporadic Parkinson’s disease.

## Materials and methods

### Study populations

To examine whether common variants in the NLRP3 inflammasome components may be associated with Parkinson’s disease, we used summary statistics from the largest Parkinson’s disease GWAS (N cases/proxy-cases= 49,053; N controls= 1,411,006)^13^. We created locus zoom plots (https://my.locuszoom.org/)^14^ for the *NLRP3*, *CASP1*, *PYCARD, IL-1*β and *IL18*, loci with +/− 500kb around each gene. We then created pathway specific PRS for the NLRP3 inflammasome using available individual level data from 14,828 Parkinson’s disease cases and 13,283 controls across 7 cohorts (detailed in Supplementary Table 1).

In our Mendelian randomization analysis, we utilized the following summary statistics datasets: Parkinson’s disease risk GWAS^13^, Parkinson’s disease age-at-onset GWAS with 17,415 cases^15^, and Parkinson’s disease progression data from GWAS studies conducted by Iwaki et al.^16^ and Tan et al.^17^. The Parkinson’s disease progression traits in the study by Iwaki et al.^16^ were measured using observational study meta-analysis of clinical scales data, we specifically used UPDRS Part III (N cases = 1,398), MMSE (N cases = 1,329), and MoCA (N cases = 1,000) scores. In the study by Tan et al.^17^, Parkinson’s disease progression was assessed using scores for motor, cognitive, and composite progression in 3,364 Parkinson’s disease patients with an average follow-up of 4.2 years.

To analyze rare variants, we performed analysis in two cohorts with available whole-exome and whole-genome sequencing data with a total of 2,943 Parkinson’s disease patients and 18,486 controls (Supplementary Table 2). Whole-genome sequencing was available from the Accelerating Medicines Partnership – Parkinson Disease (AMP-PD) initiative cohorts (https://amp-pd.org/; detailed in the Acknowledgment). Whole-exome data was available from the UK biobank (UKBB) cohort which was accessed using Neurohub (https://www.mcgill.ca/hbhl/neurohub).

### Polygenic risk score pathway analysis

In order to examine the potential genetic association of the NLRP3 complex as a whole in Parkinson’s disease (as opposed to analysis of specific SNPs), we calculated pathway PRS using PRSet for the three genes encoding the components of the NLRP3 complex (*NLRP3, PYCARD* and *CASP1*)^18^. In this analysis, we only included participants of European origin and removed first- or second-degree relatives. We further excluded all samples with sex mismatches. Only common SNPs with minor allele frequency > 0.01 and p-value < 0.05 were included in the analysis. We conducted linkage disequilibrium (LD) clumping, removing variants with r2 > 0.1 and within a 250kb distance. We performed a permutation test with 10000 repetitions to generate an empirical p-value for our gene set of interest. We used age at onset for cases, age at enrollment for controls, sex, and the top 10 principal components as covariates.

### Whole-exome and whole-genome sequencing data analysis

To determine whether rare variants in the genes encoding the components of the NLRP3 inflammasome (*NLRP3, PYCARD* and *CASP1*), we extracted genetic data from whole-exome and whole-genome sequencing datasets. Our analysis included only participants of European ancestry, and we excluded any first or second-degree relatives from the study. For whole-genome sequencing data, we performed quality control as previously described^19^. In brief, we included samples with a mean coverage of 25x and a rate of missing genotypes per sample less than 5%. For the UK Biobank’s whole-exome sequencing data, we used the Genome Analysis Toolkit (GATK, v3.8) to perform quality control. We applied the recommended filtration parameters for whole-exome sequencing data, which included a minimum depth of coverage of 10x and a minimum genotype quality (GQ) score of 20^20^. The human reference genome hg38 was used for alignment.

We analyzed the association of rare variants with minor allele frequency <0.01 using the optimized sequence kernel association test (SKAT-O)^21^. The variants were grouped to different categories: all rare variants, all non-synonymous variants, loss-of-function variants (stop, frame-shift and canonical splice-site variants) and variants with a combined annotation dependent depletion (CADD) score ≥20 (representing 1% of the top deleterious variants). To meta-analyze the two cohorts we used the metaSKAT R package^22^.

### Mendelian randomization

If modulation of the NLRP3 inflammasome is a target for therapy, then genetically driven differences in its expression, or that of the cytokines released following its activation, IL-1β and IL-18, should be causally linked to Parkinson’s disease risk or progression. To examine this possibility, we used summary-data-based Mendelian Randomization (SMR). SMR utilizes summary-level data to determine whether a causal relationship exists between an exposure and an outcome. In our specific case, we examined if differences in expression levels of the NLRP3 genes (using quantitative trait loci, QTL) are associated with risk, age-at onset and progression of Parkinson’s disease. As exposure, we used different QTL data from various studies and tissues including methylation, gene-expression and chromatin QTLs. All the QTLs we used were collected from the same resource, and we conducted analyses using SMR software developed by Yang Lab with standard settings (https://yanglab.westlake.edu.cn)^23,24^. In the present study, we used the Genotype-Tissue Expression (GTEx) project v8 release (All brain tissues, blood and liver), PsychENCODE, and BrainMeta/brain-eMeta^25–27^. As an outcome for SMR, we used the most recent Parkinson’s disease risk^13^, Parkinson’s disease age-at-onset^15^ GWASs and largest publicly available Parkinson’s disease progression GWASs^16,17^. The Bonferroni-corrected significance threshold was set at p < 0.05/185=0.00027.

### Data availability

All code is available on our GitHub repository, which can be accessed at https://github.com/gan-orlab/NLRP3. The data used in the preparation of this article were obtained from the AMP PD Knowledge Platform (https://www.amp-pd.org) and the UKBB via Neurohub (https://www.mcgill.ca/hbhl/neurohub). The full GWAS summary statistics for the 23andMe inc., discovery data set will be made available through 23andMe to qualified researchers under an agreement with 23andMe that protects the privacy of the 23andMe participants. Please visit research.23andme.com/collaborate/ for more information and to apply to access the data. QTL data and SMR software are available on the Yang Lab website (https://yanglab.westlake.edu.cn).

## Results

### No association between NLRP3 inflammasome genes and Parkinson’s disease

**Figure 1.**
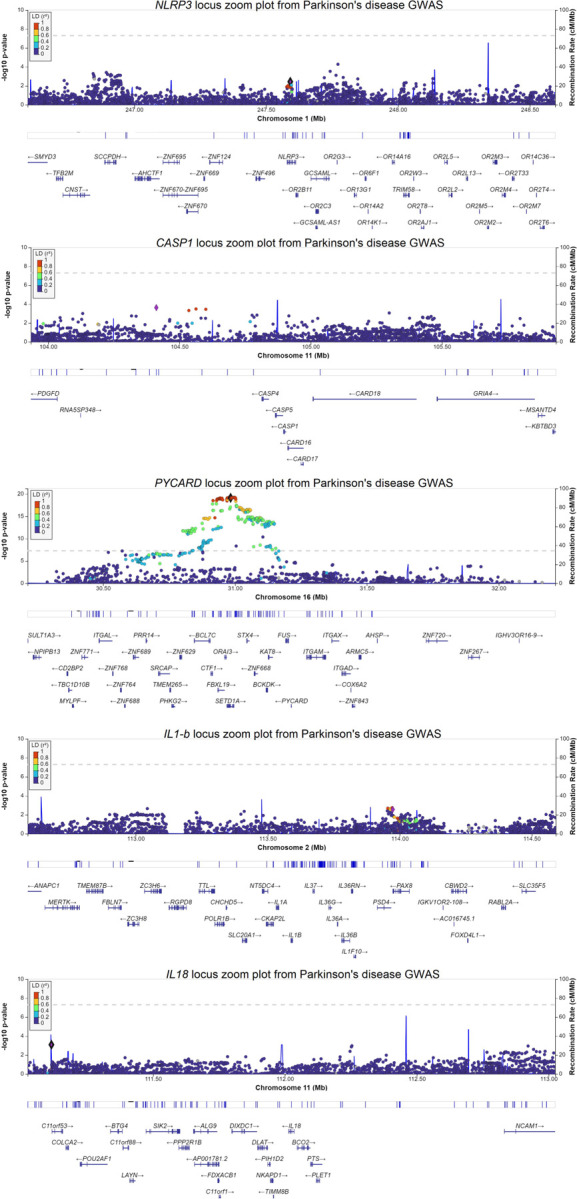
Locus zoom plot of *NLRP3*, *CASP1*, *PYCARD, IL-1*β and *IL-18* genes (+/− 500 kb) in Parkinson’s disease GWAS. The SNP with the lowest P-value in the studied gene or locus is highlighted in purple. All other SNPs are color-coded based on their r2 score in relation to the top SNP.

**Figure 2.**
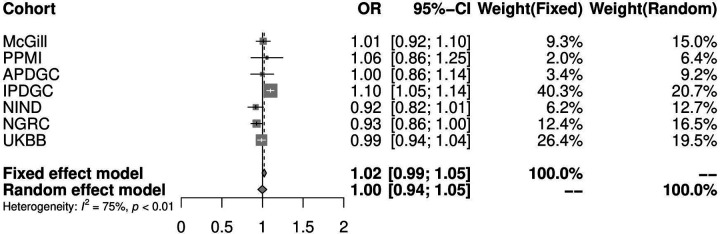
Pathway specific PRS analysis of NLRP3 inflammasome genes. OR- odds ratio; CI- confidence interval; PPMI- Parkinson’s Progression Markers Initiative; APDGC- Autopsy-Confirmed Parkinson Disease GWAS Consortium; IPDGC -International Parkinson Disease Genomics Consortium; NINDS- National Institute of Neurological Disorders and Stroke Repository Parkinson’s Disease Collection, NGRC- NeuroGenetics Research Consortium; UKBB-UK Biobank

We examined common variants from the largest available Parkinson’s disease risk GWAS^13^. We did not observe any associations between Parkinson’s disease and variants in genes composing the NLRP3 complex (*NLRP3, PYCARD* and *CASP1*) and the genes encoding the cytokines released by its activation, *IL-1*β and *IL-18* (Fig. 1). While the *PYCARD* gene is located near one of the GWAS loci (rs11150601) within *SETD1A*, *PYCARD* is just outside of the LD block, i.e. there are no variants within or in regulatory regions of *PYCARD* that are in LD (r2<0.2) with the variants that surpassed the GWAS level of significance. We then performed PRS analyses for the three NLRP3 inflammasome genes. Overall, the PRS explains a very small portion of the variance in Parkinson’s disease (1.39E-06–0.001) and was not associated with Parkinson’s disease (Fig. 2, Supplementary Table 3).

We also analyzed rare variants in two independent cohorts, including 2,943 patients and 18,486 controls, followed by a meta-analysis. We did not observe any associations between any subsets of variants in any of the genes comprising the NLRP3 inflammasome and Parkinson’s disease (Supplementary Table 4). We then performed an analysis including all the variants in all three genes combined, and in this analysis too, we did not observe any associations between rare variants and Parkinson’s disease (Supplementary Table 4).

### Mendelian randomization does not support NRLP3 as a druggable target for Parkinson’s disease.

We found that *NLRP3*, *CASP1*, *IL-1*β, *IL-18* but not *PYCARD* have been included in the database of druggable genes^28^. Nevertheless, we analyzed all five genes. Our analysis did not reveal any potential causal associations between the QTL data tested in this study and Parkinson’s disease in tissues relevant for Parkinson’s disease after correction for multiple comparisons (Supplementary Table 5).

## Discussion

Our results, using large-scale human genetic, transcriptomic and methylomic data, do not support the NLRP3 inflammasome as important in Parkinson’s disease pathogenesis or as a good target for drug development. There were no associations of common or rare variants, nor of polygenic risk score for the NLRP3 inflammasome, with risk of Parkinson’s disease. When we considered the three NLRP3 genes as druggable targets, there was no evidence that altering their expression at the RNA level may have an effect on risk, onset or Parkinson’s disease progression.

While using MR to infer efficient druggability is not a definitive test, it can still provide valuable information. For example, a recent MR study was able to replicate the beneficial effects of tumor necrosis factor (TNF) inhibition in Crohn’s disease and ulcerative colitis, and its deleterious effect in multiple sclerosis^29^. The same study also suggested that TNF inhibition might not be beneficial for Parkinson’s disease.

Understanding the role of a drug compound is essential when planning clinical trials. Studies that are not guided by genetic evidence are more likely to fail^12^. Currently, several phase 1 clinical trials targeting neuroinflammation and particularly NLRP3-inflamassome are being conducted^30^. The discordance between the hypothesis underlying these clinical trials targeting NLRP3 pathway in Parkinson’s disease and our findings suggests that efforts to target the NLRP3 inflammasome in Parkinson’s disease should be critically evaluated. It is important to select therapeutic strategies based on robust human genetic and biomarker evidence to reduce chances of trial failure. Perhaps targeting the NLRP3 inflammasome could work specifically in individuals in which this pathway is pathologically activated, but this approach is not being taken to the best of our knowledge.

Our study has several limitations that need to be acknowledged. First, we only included participants of European ancestry, which may limit the generalizability of our findings to other ethnic groups. Second, the GWASs on Parkinson’s disease progression that were used could be underpowered. Further analysis using larger datasets should be performed when they become available to confirm our findings. Finally, the SMR analysis is dependent on the quality of the expression data used for exposure, and variations in quality across datasets might influence the results.

In conclusion, our analyses do not support the involvement of the NLRP3 inflammasome in Parkinson’s disease nor its potential as a therapeutic target.

## Supplementary Material

Supplement 1 Supplementary Table 1. Study population for pathway specific PRS analysis of NLRP3 inflammasome genes
Supplementary Table 2. Study population for rare variant analysis
Supplementary Table 3. Pathway specific polygenic risk score analysis on the of NLRP3 inflammasome in Parkinson’s disease
Supplementary Table 4. Burden analysis of rare variants in NLRP3 inflammasome related genes
Supplementary Table 5. Summary based Mendelian randomization studies between NLRP3 related genes expression loci and Parkinson’s disease risk and progression

## Abbreviations:

NLRP3: nucleotide-binding oligomerization domain-, leucine-rich repeat, and pyrin domain-containing 3
CASP1: caspase-1
PRS: polygenic risk scores
MR: Mendelian randomization
IL-1β: interleukin-1 beta
IL-18: interleukin-18
GWAS: genome-wide association study
LD: linkage disequilibrium
SNP: single nucleotide polymorphism
PRSet: Polygenic Risk Score Estimation Tool
SKAT-O: sequence kernel association test optimized
AMP-PD: Accelerating Medicines Partnership – Parkinson Disease
UKBB: UK Biobank
GATK: Genome Analysis Toolkit
QTL: quantitative trait loci
SMR: summary-data-based Mendelian Randomization
GTEx: Genotype-Tissue Expression
MMSE: Mini-Mental State Examination
MoCA: Montreal Cognitive Assessment
UPDRS: Unified Parkinson’s Disease Rating Scale
CADD: combined annotation dependent depletion
